# Inflammatory myofibroblastic tumour arising in the adrenal gland: a case report

**DOI:** 10.1186/1752-1947-8-411

**Published:** 2014-12-07

**Authors:** My-Anh Tran-Dang, Neal Banga, Bernard Khoo, Alan WH Bates

**Affiliations:** 13 Department of Cellular Pathology, Royal Free Hospital, London, NW3 2QG UK; 14 Department of Endocrine Surgery, Royal Free Hospital, London, NW3 2QG UK; 15 Department of Metabolism and Experimental Therapeutics, UCL Medical School, London, NW3 2PF UK

**Keywords:** Adrenal gland, Histology, Immunohistochemistry, Inflammatory myofibroblastic tumour

## Abstract

**Introduction:**

Inflammatory myofibroblastic tumour arising in the adrenal gland is exceptional. As far as we are aware, there have been only three previous reports in the literature. We report a fourth case.

**Case presentation:**

A 29-year-old Caucasian man presented with upper quadrant pain due to a 15cm heterogenous adrenal mass that displaced his liver. He underwent an open right adrenalectomy. Histopathological examination showed the mass to be an inflammatory myofibroblastic tumour, a histologically distinctive lesion composed of myofibroblasts, plasma cells, lymphocytes and histiocytes. Ten months later he is well with no sign of recurrence.

**Conclusions:**

The lesion was indistinguishable on imaging from an adrenal cortical tumour. Surgical treatment is the same but inflammatory myofibroblastic tumour carries a favourable prognosis.

## Introduction

Inflammatory myofibroblastic tumour (IMT) is an uncommon spindle-cell proliferation that occurs at various sites including the abdomen, lungs, orbit, gastrointestinal and genitourinary tracts, usually of children and young adults [1]; however, the overall age range and anatomical distribution is wide and IMTs may arise in almost any location. The adrenal gland is a rare primary site, with only three reports in the world literature [2–4]. We report a fourth case and describe the radiological and immunohistochemical features.

## Case presentation

A 29-year-old Caucasian man presented with a 3- to 4-month history of discomfort in his right upper quadrant and right flank. He had no gastrointestinal or urinary disturbance and no endocrine symptoms. He had a previous medical history of latent tuberculosis, which was detected 3 months earlier, for which he was taking Rifanah^®^ (rifampicin and isoniazid).

**Figure 1:**
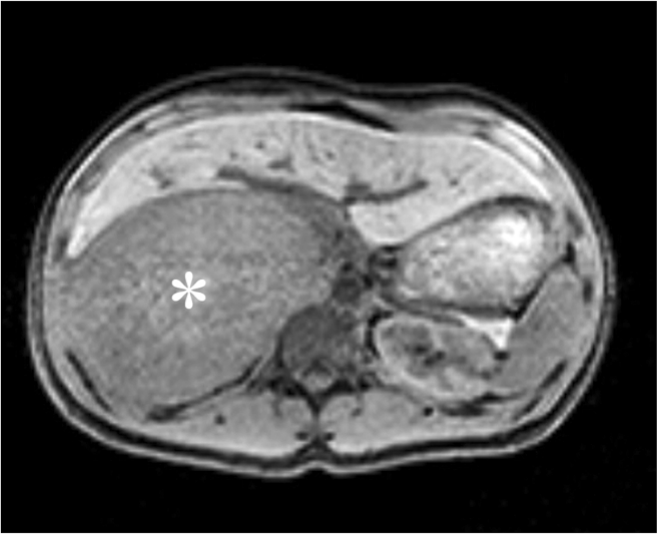
**Positron emission tomography-computed tomography axial images showing an 18×13×9cm well-defined heterogeneous hypodense lesion in the right adrenal gland (asterisk).**

On physical examination, there was anterior displacement of his liver by a palpable mass in the right hypochondrium. His blood pressure was normal. A dexamethasone suppression test, 24-hour urine catecholamine and metanephrine assays, and a plasma aldosterone: renin ratio were normal. An ultrasound scan of his abdomen and a computed tomography scan of his upper abdomen revealed a 15cm heterogenous adrenal mass compressing his liver. These appearances raised the possibility of a primary adrenal carcinoma. A metaiodobenzylguanidine (MIBG) scan and single-photon emission computed tomography scan showed no significant uptake of tracer, making a phaeochromocytoma less likely. A fluorodeoxyglucose-positron emission tomography scan revealed a right adrenal mass of size 14.5×9.5×17.2cm which was intensely metabolically active and showed dystrophic calcification and central necrosis, consistent with either adrenal cortical carcinoma or tuberculosis (Figure 1). His left adrenal gland was normal and there were no features to suggest active tuberculosis elsewhere. There was no evidence of metastatic disease. He underwent a right open adrenalectomy due to the large size of the mass. As predicted by the imaging, a massive right adrenal tumour was found adherent to his diaphragm and the posterior surface of his liver, but separate from his right kidney. It was possible to mobilize the tumour away from all of these structures without including them in the resection. The postoperative course was uneventful.

**Figure 2:**
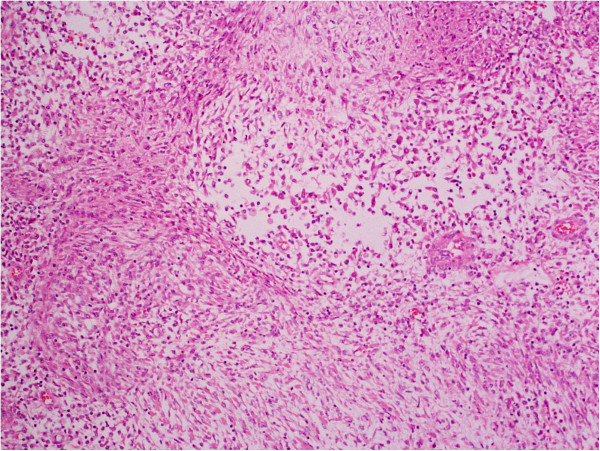
**A low-power view of the tumour showing spindled and epithelioid cells arranged predominantly in a nondescript pattern with some areas showing a fascicular and focally whorled arrangement (haematoxylin and eosin).**

**Figure 3:**
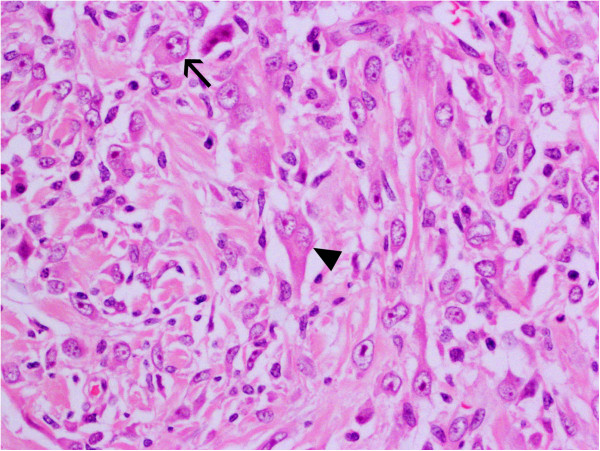
**A high-power view showing plump spindle cells with mildly atypical nuclei.** Occasional binucleated (arrowhead) and ganglion-like cells (arrow) are present.

**Figure 4:**
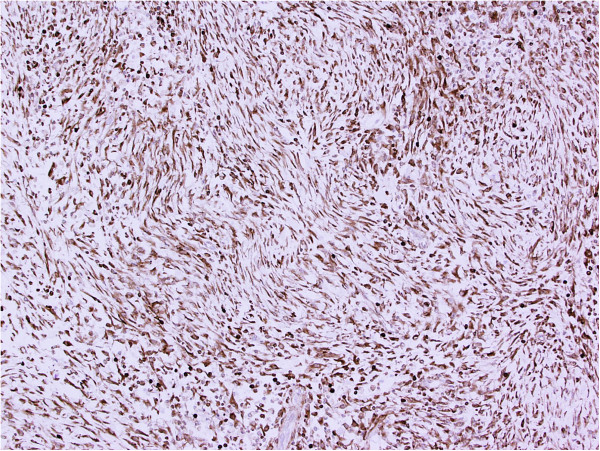
**Immunohistochemical staining for bcl-2 shows diffuse strong positivity.**

**Figure 5:**
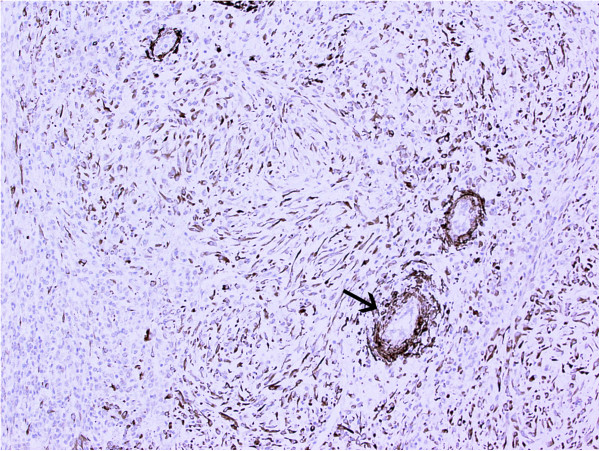
**Focal positivity for smooth muscle actin, especially around small blood vessels (arrow).**

On gross examination, the resection specimen comprised a lobulated cream-coloured tumour weighing 1.3kg and measuring 18cm in maximum dimension, with some compressed normal-looking adrenal gland at one end. The tumour had a smooth outer surface and a homogenous cut surface with a whorled appearance. There were occasional areas of haemorrhage. Microscopic examination showed a circumscribed tumour composed of plump, spindled cells and histiocytoid cells arranged haphazardly and in fascicles with intervening thick collagen bundles (Figure 2). There was an admixed moderate inflammatory cell infiltrate composed of lymphocytes, plasma cells and eosinophils. Occasional binucleated and ganglion-like cells were present (Figure 3). Mitoses were inconspicuous (<1/30 high-power fields) and no necrosis was seen. On immunohistochemical examination, the tumour cells were strongly positive for bcl-2 with focal positivity for desmin, smooth muscle actin and beta-catenin (Figures 4 and 5). Tumour cells were negative for broad-spectrum cytokeratin, epithelial membrane antigen, S100, MelanA, CD34, CD117, DOG-1 and anaplastic lymphoma kinase (ALK)-1. *MDM2* gene amplification analysis by fluorescent *in situ* hybridization was negative.

## Discussion

The term ‘inflammatory myofibroblastic tumour’ was introduced in 1986 [5] to describe a lesion previously known as plasma cell granuloma [6] or inflammatory pseudotumour [7]. The advent of immunohistochemistry brought increasing awareness of its distinctive features and potential for local recurrence [8]. In recent years, cytogenetic and molecular genetic analysis has confirmed the neoplastic nature of these lesions [9].

On histological examination, IMTs are characterized by a proliferation of plump fibroblasts or myofibroblasts accompanied by a prominent infiltrate of chronic inflammatory cells, in particular plasma cells. This feature best distinguishes IMT from fibromatosis and fasciitis, with which it overlaps. A rare epithelioid variant of IMT is associated with an abdominal location and an aggressive clinical course [10].

On immunohistochemical examination, IMTs usually express actin and may also show staining for desmin and keratin. *ALK* gene rearrangements, resulting in overexpression of ALK protein, are present in 30 to 40% of cases, mainly in the paediatric age group. Immunostaining for ALK is therefore seen only in a minority of cases. Of interest, the IMTs that have metastasized are most often ALK negative [11].

The three previous cases of adrenal IMT presented with flank pain or were incidental findings on abdominal imaging (Table 1). Clinical differential diagnoses include adrenocortical tumours, phaeochromocytoma, adrenal metastases and adrenal tuberculosis. Given that the imaging features of IMT are generally nonspecific, preoperative differentiation from other adrenal masses is very difficult. Complete surgical resection is therefore mandatory as malignancy cannot be excluded preoperatively. IMTs are usually asymptomatic, although 10 to 20% of cases are associated with pyrexia and weight loss. Tumour size at diagnosis is most often in the range of 5 to 10cm. The biological behaviour of IMT is indeterminate. After excision, 10 to 25% of patients have local recurrence. Less than 5% of cases have been reported to metastasize, but their behaviour is difficult to predict on morphological grounds, and it is increasingly believed that all cases of IMT are best regarded as low-grade sarcomas.

**Table 1:** **Summary of reported cases of adrenal myofibroblastic tumour**

Author (reference)	Patient age (years)	Sex	Presentation	Maximum tumour dimension (cm)	Recurrence
Luo *et al.*[2]	Preschool	Female	Flank pain	?	No
Wang *et al.*[3]	57	Female	Incidental	8.5	No
Chawla *et al.*[4]	20	Male	Flank pain	7	No

## Conclusions

IMT in the adrenal gland is rare but must be considered in the differential diagnosis of radiologically suspicious adrenal masses. Recurrence or metastasis of adrenal IMT has not been reported but in view of the lesion’s occasional metastatic potential at other sites careful postoperative follow up including repeat imaging at 6 months to exclude local recurrence is advised.

## Consent

Written informed consent was obtained from the patient for publication of this case report and any accompanying images. A copy of the written consent is available for review by the Editor-in-Chief of this journal.

## Abbreviations

ALK: Anaplastic lymphoma kinase
IMT: Inflammatory myofibroblastic tumour
MIBG: Metaiodobenzylguanidine.
